# Human Memory Th17 Cell Populations Change Into Anti-inflammatory Cells With Regulatory Capacity Upon Exposure to Active Vitamin D

**DOI:** 10.3389/fimmu.2019.01504

**Published:** 2019-07-17

**Authors:** Wendy Dankers, Nadine Davelaar, Jan Piet van Hamburg, Jeroen van de Peppel, Edgar M. Colin, Erik Lubberts

**Affiliations:** ^1^Department of Rheumatology, Erasmus Medical Center, Rotterdam, Netherlands; ^2^Department of Immunology, Erasmus Medical Center, Rotterdam, Netherlands; ^3^Department of Internal Medicine, Erasmus Medical Center, Rotterdam, Netherlands; ^4^Department of Rheumatology, Hospital Group Twente, Almelo, Netherlands

**Keywords:** Th17, vitamin D, Treg, rheumatoid arthritis, synovial fluid

## Abstract

Autoimmune diseases are characterized by an aberrantly activated immune system, resulting in tissue damage and functional disability in patients. An important therapeutic goal is to restore the deregulated immunological balance between pro- and anti-inflammatory T cells. This imbalance is illustrated by elevated levels and activity of memory Th17 cell populations, such as Th17, Th1/Th17, and Th17.1 cells, in various autoimmune diseases. These cells are characterized by the chemokine receptor CCR6, RORC expression and production of IL-17A, IFNγ, and TNFα. Using rheumatoid arthritis (RA) as a model of autoimmune disease, we here demonstrate that pro-inflammatory memory CCR6+ Th cells can switch into anti-inflammatory cells with regulatory capacity using the active vitamin D metabolite 1,25(OH)_2_D_3_. Memory CCR6+ Th cells, excluding Tregs, were sorted from healthy controls or treatment-naïve patients with early rheumatoid arthritis (RA) and cultured with or without 1,25(OH)_2_D_3_. Treatment with 1,25(OH)_2_D_3_ inhibited pro-inflammatory cytokines such as IL-17A, IL-17F, IL-22 and IFNγ in memory CCR6+ Th cells from both healthy controls and RA patients. This was accompanied by induction of anti-inflammatory factors, including IL-10 and CTLA4. Interestingly, these formerly pathogenic cells suppressed proliferation of autologous CD3+ T cells similar to classical Tregs. Importantly, the modulated memory cells still migrated toward inflammatory milieus *in vitro*, modeled by RA synovial fluid, and retained their suppressive capacity in this environment. These data show the potential to reset the pathogenic profile of human memory Th cells into non-pathogenic cells with regulatory capacity.

## Introduction

Autoimmune diseases are an increasing health problem in the Western industrialized countries (1). Even though treatment has improved in the last decades with the introduction of so-called “biologicals,” many patients do not respond to or become resistant to treatment. Also, we are still not able to cure these disabling diseases (2). In order to achieve better treatment of the patients, it is crucial to understand the pathogenesis of autoimmune diseases and how the important players in this pathogenesis can be modulated.

An important hallmark of many autoimmune diseases is a disturbed balance between pro- and anti-inflammatory cells, exemplified by T-helper-17 (Th17) cells and regulatory T cells (3–5). Pro-inflammatory Th17 cell populations, which are distinguished by the expression of CCR6 and include Th17, Th1/Th17, and Th17.1 cells, are elevated and more active in various autoimmune diseases including RA, systemic lupus erythematosus, and inflammatory bowel diseases (6–9). On the other hand, anti-inflammatory regulatory T cells, such as classical regulatory T cells (Tregs) and type 1 regulatory (Tr1) cells are reduced in number and functionality in human autoimmune diseases (10). Furthermore, Th17 cells and IL-17-mediated signaling are required for the development of experimental autoimmune diseases such as collagen-induced arthritis and experimental autoimmune encephalomyelitis, whereas regulatory T cells protect against experimental autoimmunity (11–15). Therefore, normalizing the Th17/Treg balance to suppress the autoimmune response is an important therapeutic goal in RA (16).

A natural agent that could play a role in the Th17/Treg balance is vitamin D. Not only are serum vitamin D levels reduced in patients with autoimmune diseases (17), its active metabolite 1,25(OH)_2_D_3_ is also a potent inhibitor of Th17 pathogenicity (18–23). Furthermore, 1,25(OH)_2_D_3_ promotes Treg differentiation and function (19, 24–26) and suppresses experimental autoimmunity (27–31).

Interestingly, it was recently shown that IL-17A-producing cells acquire a regulatory phenotype similar to Tr1 cells during normal resolution of inflammation, indicating the potential of pro-inflammatory cells to switch to a regulatory phenotype (32). Exploiting this process would be extremely relevant to restore the Th17/Treg balance in human autoimmune disease, but it is currently unknown if human Th17 cells can also transdifferentiate into Tregs and how this transition could be induced. Given its potent effects on Th17 cells and Tregs, we here studied whether human memory Th17 cell population can acquire regulatory properties using 1,25(OH)_2_D_3_.

## Materials and Methods

### Subjects

Healthy control peripheral blood mononuclear cells (PBMC, *n* = 24 in total) were used in most experiments and isolated from buffy coats obtained from Sanquin Blood Bank (Rotterdam, the Netherlands). RA PBMC, used for microarray gene expression profiles, were isolated from treatment-naïve early RA patients included in the Rotterdam Early Arthritis Cohort Study. This study was approved by the medical ethics committee of the Erasmus MC Rotterdam. RA synovial fluid was isolated from swollen knee joints of RA patients as part of usual care and informed consent was given by all patients. Relevant clinical and pharmaceutical patient information shown in Table S1.

### Cell Sorting

PBMC were isolated using a Ficoll-gradient and stored in liquid nitrogen until use. Frozen PBMC were thawed and stained with monoclonal antibodies against CD45RO (clone UCHL1), CD4 (clone RPA-T4), CD127 (clone M21), CCR6 (clone 11A9) (all BD Biosciences, San Diego, CA, USA), CD3 (clone UCHT1), CCR6 (clone GO34E2), and CD25 (clone BC96) (all Biolegend, San Diego, CA, USA) as appropriate in 0.5% BSA + 2 mM EDTA in PBS. Dead cells (typically <5% of all cells) were excluded from analysis by 4′6-Diamidino-2-Phenylindole Dilactate (DAPI). For sorting CCR6+ Th memory cells and Tregs, PBMC were pre-purified via automated magnetic-activated cell sorting (autoMACS; Miltenyi Biotec, Leiden, The Netherlands) using CD4 microbeads (Miltenyi Biotec) following manufacturer's instructions. Target cells were sorted on the FACSARIA III flow cytometer (BD Biosciences) (Figure S1).

### Cell Culture

Memory CCR6+ Th cells, excluding Tregs, (CD4+CD45RO+CCR6+CD25low/int, Figure S1) were cultured at a density of 1.25–2.5 × 10^4^ cells/ml in Iscove's Modified Dulbecco's Medium (IMDM) supplemented with 10% fetal calf serum (FCS; Gibco, Waltham, MA, USA), 100 U/ml penicillin/streptomycin, 2 mM L-glutamine (all Lonza, Verviers, Belgium), and 50 μM β-mercaptoethanol (Sigma-Aldrich, St. Louis, MO, USA). Cells were stimulated with soluble 0.3 μg/ml αCD3 and 0.4 μg/ml αCD28 (Sanquin, Amsterdam, The Netherlands) and cultured with 100 nM 1,25(OH)_2_D_3_ dissolved in 100% ethanol (Leo Pharmaceutical Products, Ballerup, Denmark) or with an equal volume of 100% ethanol (control treatment). Final ethanol concentration in medium was 0.1%.

### Suppression Assay

CD3+ cells (CD3+CD25low/intCD127+, Figure S1) were sorted as responder cells and stained with 20 μM Cell Proliferation Dye eFluor450 following manufacturer's protocol (CPD; eBioscience Inc., San Diego, CA, USA). Autologous Tregs (CD4+CD45RO+CD25hiCD127–, Figure S1) or cultured memory CCR6+ Th cells (see Cell culture) were used as putative suppressors and stained with 5 μM carboxyfluorescein succinimidyl ester (CFSE; LifeTechnologies, Eugene, OR, USA) as described by others (33). In 96-well plates 2.5 × 10^4^ responder cells were co-cultured with 2.5 × 10^4^ suppressor cells per well, under stimulation of 2.5 × 10^4^ irradiated autologous PBMC (40 Gy, RS320, X-strahl, Surrey, UK) and 10 μg/ml phytohemagglutinin P (PHA-P, Sigma-Aldrich). Where indicated, 20% cell-free synovial fluid diluted in culture medium was added. Proliferation was measured on the FACSCantoII Flow Cytometer (BD Biosciences, San Diego, CA, USA) after 6 days.

### Chemotaxis Assay

4–5 × 10^4^ cultured memory CCR6+ Th cells were seeded into the upper chamber of 96-well transwell plates with a 3.0 μm pore polycarbonate membrane (Corning, New York, NY, USA) in migration medium (T cell culture medium supplemented with 0.5% BSA instead of 10% FCS). Migration medium with or without 20% cell-free synovial fluid, 150 ng/ml CCL2, 1,000 ng/ml CCL20 or 150 ng/ml CXCL10 (all R&D Systems, Minneapolis, MN, USA) was added to the lower chamber. Each condition was run in duplicate or triplicate. After 3 h incubation at 37°C and 5% CO_2_, migrated cells were counted using CountBright beads (Invitrogen, Waltham, MA, USA) on a FACSCantoII Flow Cytometer. The migration index was calculated by the number of cells that migrated in response to chemokines or synovial fluid divided by the number of cells that migrated to migration medium.

### Flow Cytometry

For flow cytometry after culture, cells were stained in FACS buffer (0.5% BSA + 0,05% NaN3 in PBS) with monoclonal antibodies against CD4, CCR3, CXCR3, CCR2, CXCR4 (BioLegend), CCR6 (R&D Systems), CD45RO, CCR6, CCR7 (BD Biosciences), and CXCR5 (eBioscience). Samples were measured on a FACSCantoII Flow Cytometer.

### Microarray

RNA was isolated from cells using the GenElute Mammalian Total RNA Miniprep Kit (Sigma-Aldrich) following manufacturer's instructions. RNA purity was measured using the 2100 Bioanalyzer Instrument (Agilent, Santa Clara, CA, USA). For each sample 150 ng total RNA was amplified using the Illumina TotalPrep RNA Amplification Kit (Ambion, Invitrogen). Seven hundred fifty nanogram of amplified RNA was hybridized following the Illumina protocol. Gene expression profiles were generated using HumanHT-12 v4 BeadChip human whole-genome expression arrays, which were scanned using iScan (all Illumina, San Diego, CA, USA). Raw data were background subtracted using Illumina GenomeStudio (V2010.1, Illumina) and used for further analysis. The probe numbers of genes discussed in this paper are listed in Table S2. The microarray data are deposited in the GEO repository under accession code GSE133426.

### ELISA

Concentrations of IL-17A, IL-17F, IL-22, IFNγ, and IL-10 were measured in culture supernatant using Ready-SET-Go! ELISA (eBioscience) according to manufacturer's instructions.

### RT-PCR

RNA was isolated using the GenElute Mammalian Total RNA Miniprep Kit (Sigma-Aldrich) following manufacturer's instructions. Isolated RNA was treated with 0.1 U/μl DNAse (Invitrogen) and reverse transcribed into cDNA using 10 U/μl Superscript II (Invitrogen) and random hexamer primers. Primers were designed using ProbeFinder Software and probes were selected from the Universal Probe Library (Roche Applied Science, Indianapolis, IN, USA) (Table S3). Real-time PCR was performed using the Viia7 system (Applied Biosystems, Waltham, MA, USA) and data were analyzed using QuantStudio Real-Time PCR Software version 1.3 (Applied Biosystems). Gene expression was normalized against hypoxanthine phosphoribosyltransferase (HPRT).

### Statistical Analysis

Paired Student's *T*-test or ANOVA with a Bonferroni post-test were used to test differences between two or more groups, respectively. The analysis was performed using Prism software version 6.01 (GraphPad Software, La Jolla, CA, USA).

## Results

### 1,25(OH)_2_D_3_ Inhibits the Th17 Phenotype While Inducing a Regulatory Gene Signature in Memory CCR6+ Th Cells

**Figure 1:**
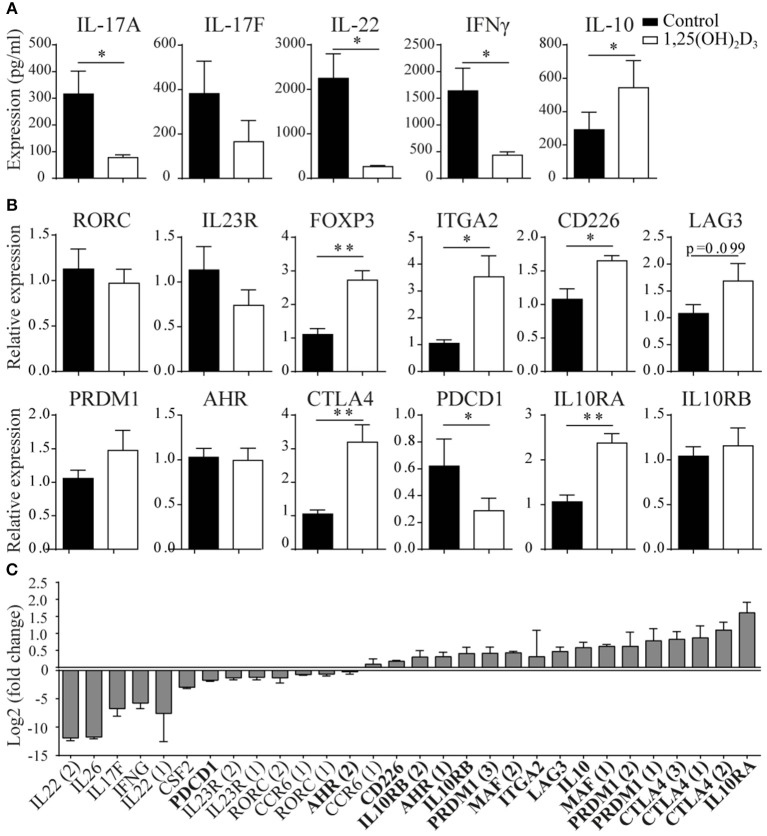
1,25(OH)_2_D_3_ treatment reduces pro-inflammatory gene expression in memory CCR6+ Th cells, while inducing an anti-inflammatory expression profile. Memory CCR6+ Th cells were sorted from healthy controls **(A,B)** or treatment-naïve early RA patients **(C)** and cultured for 3 days with or without 1,25(OH)_2_D_3_. Cytokine expression was measured using ELISA **(A)** and gene expression via RT-PCR **(B)** or microarray-based gene-expression profiles **(C)**. In **(C)**, fold change is calculated by dividing the expression level after 1,25(OH)_2_D_3_ treatment by the level after control treatment. Pro-inflammatory genes are in normal font, anti-inflammatory genes in bold font. FOXP3 and IL-17A were investigated but could not be detected by the probes on the microarray. Numbers behind genes indicate different probes that were used to measure gene expression. Mean and SEM are given for 3 patients **(C)** or 7–8 healthy controls pooled from 2 independent experiments **(A,B)**, ^*^p < 0.05, ^**^p < 0.01.

To study the potential conversion from a pro- to an anti-inflammatory phenotype in memory CCR6+ Th cells, we first studied their cytokine expression profiles after *in vitro* 1,25(OH)_2_D_3_ treatment. Previously, we have shown that 1,25(OH)_2_D_3_ already has inhibitory effects on Th17 cells from a concentration of 0.1 nM, but that a dose of 100 nM has the optimal suppressive effects on their pro-inflammatory phenotype (22, 34). Therefore, memory CCR6+ Th cells, specifically excluding Tregs since they can also express CCR6 (35), were sorted from healthy controls and cultured with or without 100 nM 1,25(OH)_2_D_3_ for 3 days. In line with previous data (18), we found that 1,25(OH)_2_D_3_ inhibited the pro-inflammatory cytokines IL-17A, IL-22, and IFNγ. Interestingly, the anti-inflammatory cytokine IL-10 was upregulated (Figure 1A). This potential induction of an anti-inflammatory phenotype was further studied via gene expression analyses for factors important for the Th17 phenotype and pathogenicity (RORC, IL23R), transcription factors characterizing Treg or Tr1 cells (FOXP3, ITGA2, CD226, LAG3) (36, 37), genes required for Tr1 development and IL-10 production (PRDM1, AHR, MAF) (38–40) and factors important for the suppressive function of Tregs and Tr1 cells (CTLA4, PDCD1, IL10RA, IL10RB) (41–43). Although RORC and IL23R were not significantly modulated in the memory CCR6+ Th cells from healthy controls, regulatory genes such as FoxP3 and CTLA4 were induced in response to 1,25(OH)_2_D_3_ (Figure 1B). While MAF showed a trend toward induction by 1,25(OH)_2_D_3_, it was only expressed in two out of seven donors (data not shown). Of the other investigated genes only IL10RB, PRDM1, and AHR were not modulated by 1,25(OH)_2_D_3_, whereas PDCD1, encoding for the inhibitory co-receptor PD1, was inhibited (Figure 1B).

These data show that 1,25(OH)_2_D_3_ induces an anti-inflammatory signature in CCR6+ cells from healthy controls, but this effect may not be similar in the more activated cells from patients with an autoimmune disease such as rheumatoid arthritis (RA) (7, 22). Therefore, memory CCR6+ Th cells from treatment-naïve early RA patients were treated with or without 1,25(OH)_2_D_3_ and used to generate microarray-based gene expression profiles. Similar to our findings in healthy cells, 1,25(OH)_2_D_3_ inhibited pro-inflammatory genes in the memory CCR6+ Th cells from RA patients, except that in RA cells also RORC and IL23R are inhibited (Figure 1C, normal font). Interestingly, also in the RA cells the anti-inflammatory genes were upregulated, again with the exception of PDCD1 (Figure 1C, bold font).

Altogether, these data suggest that 1,25(OH)_2_D_3_ inhibits the pro-inflammatory phenotype of pathogenic memory CCR6+ Th cells, while inducing an anti-inflammatory phenotype in these cells.

### 1,25(OH)_2_D_3_-Treated Memory CCR6+ Th Cells Suppress Proliferation of CD3+ T Cells

**Figure 2:**
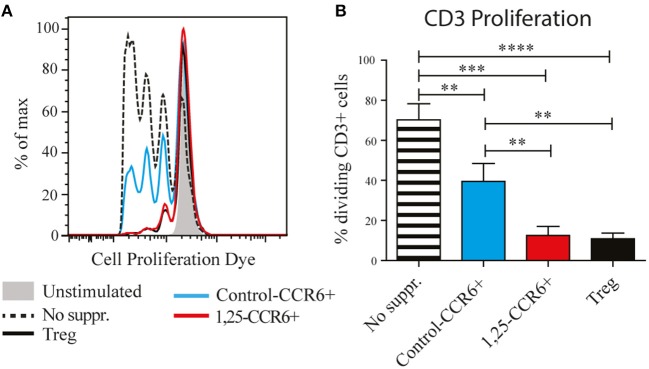
Memory CCR6+ Th cells acquire regulatory capacities after treatment with 1,25(OH)_2_D_3_. Memory CCR6+ Th cells (without regulatory T cells) were sorted from healthy controls and cultured for 3 days with or without 1,25(OH)_2_D_3_. Then they were co-cultured for 6 days with CPD-stained CD3+ T cells to assess their suppressive potential. Target cell proliferation was assessed using flow cytometry, and sorted Treg cells were used as a positive control for suppression of proliferation. **(A)** Representative plot of proliferation data. **(B)** Pooled proliferation data for 3 healthy donors. No suppr; no suppressors. Control-CCR6+; control-treated memory CCR6+ Th cells. 1,25-CCR6+; 1,25(OH)_2_D_3_-treated memory CCR6+ Th cells. Mean and SEM are given and are representative of 4 independent experiments, ^**^p < 0.01, ^***^p < 0.001, ^****^p < 0.0001.

The induction of genes such as IL-10 and CTLA4, which are required for the suppressive function of regulatory T cells, suggests that 1,25(OH)2D3-treated memory CCR6+ Th cells could also have suppressive properties. Therefore, their capacity to suppress proliferation of autologous CD3+ T cells (excluding Tregs) was compared with the suppressive potential of classical Tregs. In these suppression assays, control-treated memory CCR6+ Th cells already slightly suppressed proliferation of CD3+ T cells (Figure 2), a phenomenon that has been described before when untreated cultured T cells are used as suppressor cells (24). Importantly, 1,25(OH)_2_D_3_-treated memory CCR6+ Th cells were equally potent suppressors of CD3+ T cell proliferation as Tregs (Figure 2). This was similar for the CD4+ cells and the CD4– cells within the CD3+ T cells, demonstrating that both Th cells and cytotoxic T cells, which are the main CD3+CD4– population, can be suppressed by treated CCR6+ T helper memory cells (Figure S2). These data show that the pro-inflammatory memory CCR6+ Th cells acquire regulatory properties upon treatment with 1,25(OH)_2_D_3_.

### 1,25(OH)_2_D_3_-Treated Memory CCR6+ Th Cells Still Migrate Toward Inflammatory Milieus *in vitro*

**Figure 3:**
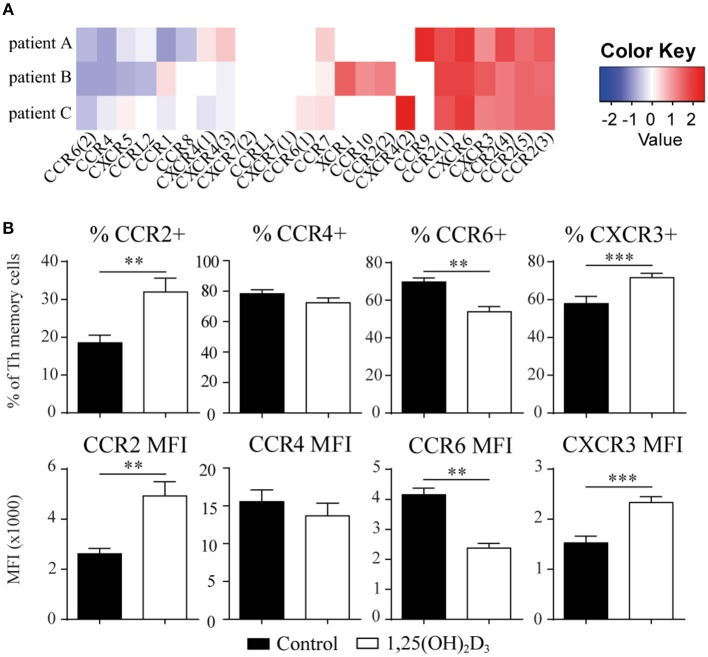
The chemokine receptor profile in memory CCR6+ Th shifts upon treatment with 1,25(OH)_2_D_3_. **(A)** Memory CCR6+ Th cells were sorted from treatment-naïve early RA patients and cultured for 3 days with or without 1,25(OH)_2_D_3_. RNA was isolated and used for microarray analysis. Log2 of fold change induced by 1,25(OH)_2_D_3_ treatment is shown for all chemokine receptors that were detected in the microarray data. **(B)** Changes in the chemokine receptor profile were verified using flow cytometry on memory CCR6+ Th cells sorted from healthy controls which were cultured for 3 days with or without 1,25(OH)_2_D_3_. Mean and SEM are given for 3 patients **(A)** or 5 healthy controls **(B)**. MFI; mean fluorescent intensity, ^**^p < 0.01, ^***^p < 0.001.

**Figure 4:**
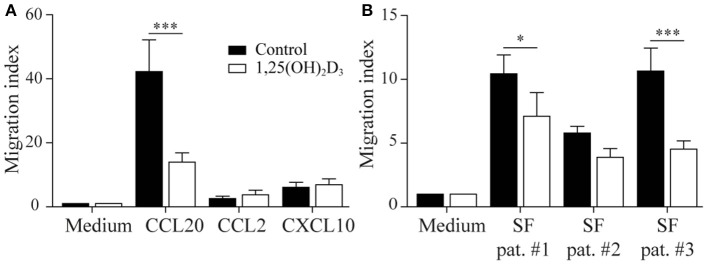
1,25(OH)_2_D_3_-treated memory CCR6+ Th cells still migrate toward an inflammatory environment. Memory CCR6+ Th cells were sorted from healthy controls and cultured for 3 days with or without 1,25(OH)_2_D_3_. Their migratory capacity toward CCL20, CXCL10, CCL2 **(A)** or 20% synovial fluid from RA patients **(B)** was assessed using a 3-μm pore transwell system. Mean and SEM are shown for 5 healthy controls from 2 independent experiments, ^*^p < 0.05, ^***^p < 0.001.

We next asked whether the 1,25(OH)_2_D_3_-treated memory CCR6+ Th cells could contribute to the suppression of synovial inflammation, for which the cells need to be able to migrate toward inflammation. Therefore, the effect of 1,25(OH)_2_D_3_ on the chemokine receptors expressed in treatment-naïve early RA patients was first investigated using the microarray-based gene expression profiles. The 1,25(OH)2D3-exposed memory CCR6+ Th cells showed a shift in the chemokine receptor profile, where some receptors are upregulated while others are downregulated (Figure 3A). Importantly, the signature chemokine receptor CCR6 is inhibited by 1,25(OH)_2_D_3_, while other receptors such as CXCR3 and CCR2 are upregulated. These data were verified on protein level using healthy memory CCR6+ Th cells, where 1,25(OH)2D3 similarly modulates the expression of all chemokine receptors except CCR4 (Figure 3B; Figure S3). To investigate what these changes mean for the actual migration of memory CCR6+ Th cells, both untreated and 1,25(OH)2D3-treated memory CCR6+ Th cells were tested for their migration toward CCL20, CXCL10, and CCL2. These chemokines were selected because they are the ligands for CCR6, CXCR3, and CCR2, respectively, which were the most up- or downregulated chemokine receptors in both healthy and RA cells. In a transwell chemotaxis assay, migration of memory CCR6+ Th cells toward CCL20, the ligand for CCR6, was significantly decreased after treatment with 1,25(OH)_2_D_3_ (Figure 4A). On the other hand, there was no difference in the migratory capacity of the cells toward ligands for CXCR3 and CCR2, CXCL10, and CCL2 respectively, (Figure 4A). Since single chemokines may not accurately represent the site of inflammation, the migratory potential toward synovial fluid obtained from RA patients was assessed as an example of an inflammatory environment. Twenty percent synovial fluid from these patients, all with a high disease activity (Table S1), induced migration of both control-treated and 1,25(OH)_2_D_3_-treated cells, although the latter migrated less efficiently in two out of three patients (Figure 4B). This indicates 1,25(OH)_2_D_3_-treated memory CCR6+ Th cells can still migrate toward synovial fluid *in vitro*, although their migratory capacities may be slightly altered.

### 1,25(OH)_2_D_3_-Treated Memory CCR6+ Th Cells Are Equally Suppressive as Tregs in Synovial Fluid

**Figure 5:**
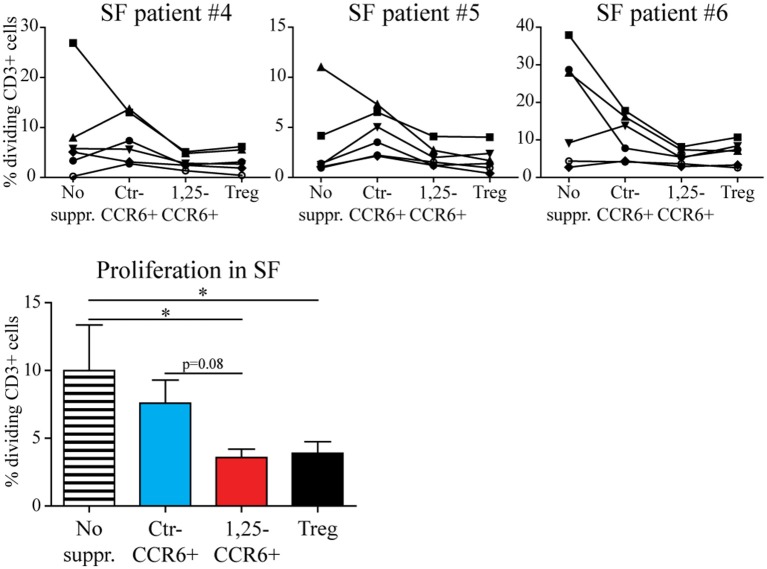
The suppressive capacity of 1,25(OH)_2_D_3_-treated memory CCR6+ Th cells is retained in the presence of RA synovial fluid. Memory CCR6+ Th cells were sorted from healthy controls and cultured for 3 days with or without 1,25(OH)_2_D_3_. They were then used in the same suppression assay as for Figure 3, but in the presence of 20% synovial fluid. Proliferation for CD3+ T cells of six healthy donors (each depicted by its own symbol) is shown with or without suppressor cells, in the presence of three different synovial fluid samples (upper panel). Overall proliferation in synovial fluid is calculated from the average proliferation of T cells from each healthy donor in three different synovial fluids (lower panel). No suppr; no suppressors. Ctr-CCR6+; control-treated memory CCR6+ Th cells. 1,25-CCR6+; 1,25(OH)_2_D_3_-treated memory CCR6+ Th cells. Mean and SEM are shown for 6 healthy controls from 2 independent experiments. ^*^p < 0.05.

Although memory CCR6+ Th cells acquire a functional regulatory phenotype and migrate toward sites of inflammation upon exposure, they can only contribute to inhibiting synovial inflammation if the local pro-inflammatory conditions do not reverse the 1,25(OH)_2_D_3_-induced changes. Therefore, the suppressive capacity of 1,25(OH)2D3-modulated memory CCR6+ Th cells was assessed in the presence of 20% RA synovial fluid using a similar assay as for Figure 2. Although T cell proliferation was lower in synovial fluid than in medium (see also Figure 2), 1,25(OH)_2_D_3_-treated memory CCR6+ Th cells still suppressed T cell proliferation similar to Tregs under these inflammatory conditions (Figure 5).

## Discussion

In this study, we demonstrated that human pro-inflammatory IL-17A-producing memory CCR6+ Th cells acquire an anti-inflammatory gene expression profile and regulatory properties upon exposure to 1,25(OH)_2_D_3_. Importantly, these modulated cells can still migrate toward synovial fluid and maintain their suppressive capacity in this pro-inflammatory environment.

The 1,25(OH)_2_D_3_-mediated inhibition of the pro-inflammatory gene signature in memory CCR6+ Th cells is accompanied by induction of an anti-inflammatory gene signature in both healthy controls and treatment-naïve early RA patients. An exception to this pattern was the inhibition of the suppressive co-receptor PD-1 by 1,25(OH)_2_D_3_. Although not much research has been performed into the role of PD-1 in RA, one case report suggests that PD-1 may be less important in suppressing the autoimmune response than CTLA4. In this case, a RA patient suffering from metastatic melanoma experienced RA flares when treated with anti-CTLA4, but not when treated with anti-PD-1 (44). Therefore, the inhibition of PD-1 by 1,25(OH)_2_D_3_ may also not be important for the regulatory characteristics of modulated CCR6+ Th cells.

Although the pattern of gene modulation by 1,25(OH)_2_D_3_ was largely similar between healthy controls and RA patients, there were a few notable exceptions. Firstly, RORC and IL23R were inhibited by 1,25(OH)_2_D_3_ in the cells from RA patients, but not from healthy controls. Also, MAF was expressed in cells from treatment-naïve early RA patients, but was hardly detectable in healthy control cells. These differences could be due to the higher activation state of the memory CCR6+ Th cells from RA patients (7, 22). Furthermore, since vitamin D is considered to be immunomodulatory rather than immunosuppressive (17), its effects on highly activated RA cells may be stronger than on healthy cells. The exact mechanism underlying the difference in modulation between healthy cells and cells from RA and other autoimmune diseases is of great interest for future research and could provide insight into disease-specific pathogenesis.

In line with the expression of anti-inflammatory genes, 1,25(OH)_2_D_3_-treated memory CCR6+ Th cells suppress proliferation of autologous T cells. This data corresponds with the murine immune system, where IL-17A-producing cells acquire Tr1-like properties during resolution of inflammation (32). In human cells, an analog of 1,25(OH)_2_D_3_ induces a regulatory phenotype in the total CD4+ population (Tregs excluded) (45). Also, 1,25(OH)_2_D_3_ in combination with IL-2 can promote this phenotypical change (24). However, both studies still include naïve T cells, in which IL-10 production is also induced upon 1,25(OH)_2_D_3_ treatment (46). Our study is the first to demonstrate that regulatory properties can be induced in a population of highly purified human pro-inflammatory memory Th cells.

Despite the regulatory capacity of the 1,25(OH)_2_D_3_-treated memory CCR6+ Th cells, the exact phenotype of these cells remains to be determined. We verified protein surface expression of important regulatory surface markers using flow cytometry and found that the MFI of PD1, TIM3 and LAG3 were all significantly lower after exposure to 1,25(OH)2D3 in contrast to CTLA4 and CD49B (data not shown). Further characterization of the transcriptomic and proteomic changes induced by 1,25(OH)2D3 in these cells is needed to further identify the phenotype of these anti-inflammatory cells with regulatory capacity.

For the conversion from a pro-inflammatory IL-17A-producing memory CCR6+ Th cell to a regulating cell to be functional in suppressing autoimmune diseases, the cells should migrate toward the site of inflammation. 1,25(OH)_2_D_3_ treatment modulates the chemokine receptor profile of memory CCR6+ Th cells, which alters their migration toward CCL20 but not CXCL10 and CCL2. This is in line with previous studies, which reported altered T cell migration toward various chemokines after 1,25(OH)_2_D_3_ treatment (20, 45). The migration capacity of 1,25(OH)_2_D_3_-treated memory CCR6+ Th cells toward RA synovial fluid was unaffected or partially reduced, in a patient-dependent manner. The differences between patients observed in our study could be treatment-related, since for example CCL2 is inhibited by TNFα treatment (47, 48), and CXCL9 and CXCL10 decrease during treatment response (49). Furthermore, the synovial fluid and memory CCR6+ Th cells were isolated from allogeneic donors, which may play a role in our chemotaxis assays. However, since the modulated memory CCR6+ Th cells still migrated faster toward synovial fluid than to medium alone, we concluded they still migrate toward the site of inflammation.

The modulated cells cannot only migrate to RA synovial fluid, they also still suppress autologous T cell proliferation in this environment. Notably, the baseline proliferation of T cells was already inhibited in the presence of synovial fluid. This might be due to the viscosity of the synovial fluid, which complicates the cell-cell interaction that is required for stimulation by irradiated PBMC. Inhibition of T cell proliferation has been shown before, but only when more than 50% of the medium consisted of synovial fluid and under stimulation that is not dependent on cell-cell interaction (50). Nonetheless, the novel finding in this study that 1,25(OH)_2_D_3_-treated memory CCR6+ Th cells retain their suppressive capacity in synovial fluid suggests that these cells may have therapeutic value in autoimmune diseases such as RA.

It is tempting to consider transfer of autologous 1,25(OH)_2_D_3_-treated memory CCR6+ Th cells to promote tolerance, similar to the 1,25(OH)_2_D_3_-treated dendritic cells that are currently under clinical investigation (51). This could be beneficial not only for RA, but also other Th17-driven autoimmune diseases. For such a treatment to be effective, the cells require a stable anti-inflammatory phenotype and migratory capacity toward the site of inflammation. Importantly, the finding that modulated memory CCR6+ Th cells suppress T cell proliferation not only upon restimulation with irradiated PBMC and PHA, an inevitable consequence of the experimental setup, but also in the presence of synovial fluid, indicates that the anti-inflammatory phenotype might be stable. On the other hand, RORC is still expressed in 1,25(OH)_2_D_3_-treated memory CCR6+ Th cells in both healthy controls and RA patients, suggesting that the cells did not completely transdifferentiate into a classical Treg. Therefore, the extent of the phenotype stability after 1,25(OH)_2_D_3_ exposure is of great clinical interest for various autoimmune diseases. Both migration capacity and therapeutic efficacy of 1,25(OH)_2_D_3_-treated memory CCR6+ Th cells should be further determined using transfer experiments in autoimmune models.

Another issue that should be taken into account for the clinical translation of these findings is the dose of 1,25(OH)_2_D_3_ used in this study. Physiological steady-state levels are thought to be around 0.1–0.2 nM (52, 53), whereas we used a higher dose of 100 nM. However, the level of 1,25(OH)_2_D_3_ might be higher at the site of inflammation due to the capability of immune cells to convert inactive vitamin D into the active 1,25(OH)_2_D_3_ metabolite (54, 55). Currently this issue remains unresolved and direct supplementation of active 1,25(OH)_2_D_3_ is not possible due to the possibility of severe side effects. Therefore, it will be of great interest to understand the mechanism underlying the shift in phenotype in response to 1,25(OH)_2_D_3_ as this may provide us with new therapeutic targets. As a first step to understanding the mechanism, the various subpopulations within the memory CCR6+ Th cells should be investigated. memory CCR6+ Th cells are a heterogeneous mixture of pro-inflammatory cells, such as Th17, Th1/Th17 and Th17.1 cells, but also unclassified cells that do not seem to belong to either of these populations. All CCR6+ subpopulations express RORC and varying levels of IL-17A, but they differ in chemokine receptor expression, production of other cytokines such as IFNγ and GM-CSF and transcription factor expression (e.g., Tbx21) (56). It is of great interest to investigate which of these subpopulations is more prone to switch to an anti-inflammatory cell with regulatory capacity, so certain cell types can be more specifically targeted.

In this study it was demonstrated that committed pro-inflammatory IL-17A-producing memory CCR6+ Th cells acquire regulatory properties upon treatment with 1,25(OH)_2_D_3_. This process could normalize the balance between pro- and anti-inflammatory mediators in autoimmune diseases. These data provide a basis to further explore the mechanisms to reset the pathogenic profile of memory T cells during autoimmune inflammatory diseases. This may lead to new developments to activate resolution of inflammation in RA and other autoimmune diseases.

## Data Availability

The microarray data are deposited in the GEO repository under accession code GSE133426.

## Ethics Statement

This study was carried out in accordance with the recommendations of the medical ethics committee of the Erasmus MC Rotterdam, The Netherlands with written informed consent from all subjects. All subjects gave written informed consent in accordance with the Declaration of Helsinki. The protocol was approved by the medical ethics committee of the Erasmus MC Rotterdam, The Netherlands.

## Author Contributions

WD contributed to the study design, performed experiments and wrote the manuscript. ND performed experiments and revised the manuscript. JvH performed experiments and revised the manuscript. JvdP performed the microarray experiments, performed initial analyses and revised the manuscript. EC contributed to the study design and revised the manuscript. EL designed the study and revised the manuscript.

### Conflict of Interest Statement

The authors declare that the research was conducted in the absence of any commercial or financial relationships that could be construed as a potential conflict of interest.
