# Supplementary material for: Human Memory Th17 Cell Populations Change Into Anti-inflammatory Cells With Regulatory Capacity Upon Exposure to Active Vitamin D
Source: Front Immunol. 2019 Jul 17;10:1504. doi: 10.3389/fimmu.2019.01504 (PMC6651215; doi:10.3389/fimmu.2019.01504)
Supplement: Supplementary file 1 [file Table_1.pdf]

Parameters	Treatment-naïve early RA patients (PBMC) Mean ± SEM (n=3)	Established RA patients (synovial fluid) Mean ± SEM (n=6)
Age	42.06 ± 8.00	58.17 ± 2.14
DAS28	2.59 ± 0.15	4.57 ± 0.29
VAS	42.33 ± 5.89	81.50 ± 8.32
ESR	30.00 ± 2.62	26.40 ± 8.57
CRP	12.33 ± 8.03	19.00 ± 2.87
TJC28	2.33 ± 0.72	3.33 ± 0.51
SJC28	3.67 ± 0.98	1.83 ± 0.44
Disease duration (days)	99.00 ± 28.58	N/A
Medication	None	Methotrexate (5/6)<br>Vitamin D (800 IU/day, 4/6)<br>Tocilizumab (1/6)<br>Adalimumab (2/6)<br>Conventional DMARDs (1/6)

Table S1 Characteristics of patients used in this study. PBMC of treatment-naïve early RA patients were used for microarray gene expression profiles
