# Supplementary material for: Human Memory Th17 Cell Populations Change Into Anti-inflammatory Cells With Regulatory Capacity Upon Exposure to Active Vitamin D
Source: Front Immunol. 2019 Jul 17;10:1504. doi: 10.3389/fimmu.2019.01504 (PMC6651215; doi:10.3389/fimmu.2019.01504)
Supplement: Supplementary file 2 [file Table_2.pdf]

| Gene | Probe ID | Gene | Probe ID |
|---|---|---|---|
| AHR (1) | ILMN_1812640 | CXCR5 | ILMN_2337928 |
| AHR (2) | ILMN_2162799 | CXCR6 | ILMN_1674640 |
| CCR1 | ILMN_1678833 | CXCR7 (1) | ILMN_1798360 |
| CCR10 | ILMN_1666493 | CXCR7 (2) | ILMN_2371458 |
| CCR2 (1) | ILMN_1774761 | FOXP3 | ILMN_1768049 |
| CCR2 (2) | ILMN_2276996 | IFNG | ILMN_2207291 |
| CCR2 (3) | ILMN_1777461 | IL10 | ILMN_2073307 |
| CCR2 (4) | ILMN_1669062 | IL10RA | ILMN_1652825 |
| CCR2 (5) | ILMN_1769895 | IL10RB (1) | ILMN_1767360 |
| CCR4 | ILMN_2086143 | IL10RB (2) | ILMN_2230892 |
| CCR6 (1) | ILMN_1690907 | IL17A | ILMN_1774983 |
| CCR6 (1) | ILMN_2387696 | IL17F | ILMN_2188247 |
| CCR6 (2) | ILMN_2387696 | IL22 (1) | ILMN_1735208 |
| CCR6 (2) | ILMN_1690907 | IL22 (2) | ILMN_2100046 |
| CCR7 | ILMN_1715131 | IL23R (1) | ILMN_1734937 |
| CCR8 | ILMN_1739421 | IL23R (2) | ILMN_2223663 |
| CCR9 | ILMN_1664316 | IL26 | ILMN_2123182 |
| CCRL1 | ILMN_1773992 | ITGA2 | ILMN_1665792 |
| CCRL2 | ILMN_3190833 | LAG3 | ILMN_1813338 |
| CD226 | ILMN_1687825 | MAF (1) | ILMN_1722206 |
| CSF2 | ILMN_1661861 | MAF (2) | ILMN_1719543 |
| CTLA4 (1) | ILMN_2261627 | PDCD1 | ILMN_1806725 |
| CTLA4 (2) | ILMN_2348905 | PRDM1 (1) | ILMN_2414165 |
| CTLA4 (3) | ILMN_1763487 | PRDM1 (2) | ILMN_2298159 |
| CXCR3 | ILMN_1797975 | PRDM1 (3) | ILMN_1655077 |
| CXCR4 (1) | ILMN_2320888 | RORC (1) | ILMN_1734366 |
| CXCR4 (2) | ILMN_2246410 | RORC (2) | ILMN_1771126 |
| CXCR4 (3) | ILMN_1801584 | XCR1 | ILMN_1764034 |

**Table S2** Illumina probe ID and the corresponding gene code as used in this report.
