# Supplementary material for: Human Memory Th17 Cell Populations Change Into Anti-inflammatory Cells With Regulatory Capacity Upon Exposure to Active Vitamin D
Source: Front Immunol. 2019 Jul 17;10:1504. doi: 10.3389/fimmu.2019.01504 (PMC6651215; doi:10.3389/fimmu.2019.01504)
Supplement: Supplementary file 3 [file Table_3.pdf]

Gene	Forward primer	Reverse primer	Probe
HPRT	5’-TGACCTTGATTTATTTTGCATACC-3’	5’-CGAGCAAGACGTTCAGTCCT-3’	73
FOXP3	5’-ACCTACGCCACGCTCATC-3’	5’-TCATTAAGTGTCCGCTGCT-3’	50
CTLA4	5’-TCACAGCTGTTTCTTTGAGCA-3’	5’-AGGCTGAAATTGCTTTTCACA-3’	21
CD49B	5’-TCGTGCACAGTTTTGAAGATG-3’	5’-TGGAACACTTCCTGTTGTTACC-3’	7
LAG3	5’-CAAGAACGCTTTGTGTGGAG-3’	5’-CCAGCCAAGGTCCTGAGA-3’	82
IL10RA	5’-CCGAAAGAAGCTACCCAGTG-3’	5’-GGACGCTGGCTGATGAAG-3’	23
IL10RB	5’-GGTCGTGTGCTTGGAGGA-3’	5’-GGTACCATTCCCAATGCTGA-3’	20
CD226	5’-CGTGATGAGATTGACTGTAGCC-3’	5’-AACAAACAACAACAATAAAACTGTCC-3’	5
PRDM1	5’-ACGTGTGGGTACGACCTTG-3’	5’-CTGCCAATCCCTGAAACCT-3’	67
PDCD1	5’-AGAAGGCGGCACTCTGGT-3’	5’-GGCCTGTCTGGGGAGTCTA-3’	32
AHR	5’-AGCCGGTGCAGAAAACAGT-3’	5’-CTATGCCGCTTGGAAGGAT-3’	33
RORC	5’-CAGCGCTCCAACATCTTCT-3’	5’-CCACATCTCCCACATGGACT-3’	69
IL23R	5’-CCTGGCTCTGAAGTGGAATTA-3’	5’-GGCTATTACTGCATCCCATTG-3’	38

Table S3 Primers and probes used for RT-PCR
