# Supplementary material for: Human Memory Th17 Cell Populations Change Into Anti-inflammatory Cells With Regulatory Capacity Upon Exposure to Active Vitamin D
Source: Front Immunol. 2019 Jul 17;10:1504. doi: 10.3389/fimmu.2019.01504 (PMC6651215; doi:10.3389/fimmu.2019.01504)
Supplement: Supplementary file 4 [file Image_1.pdf]

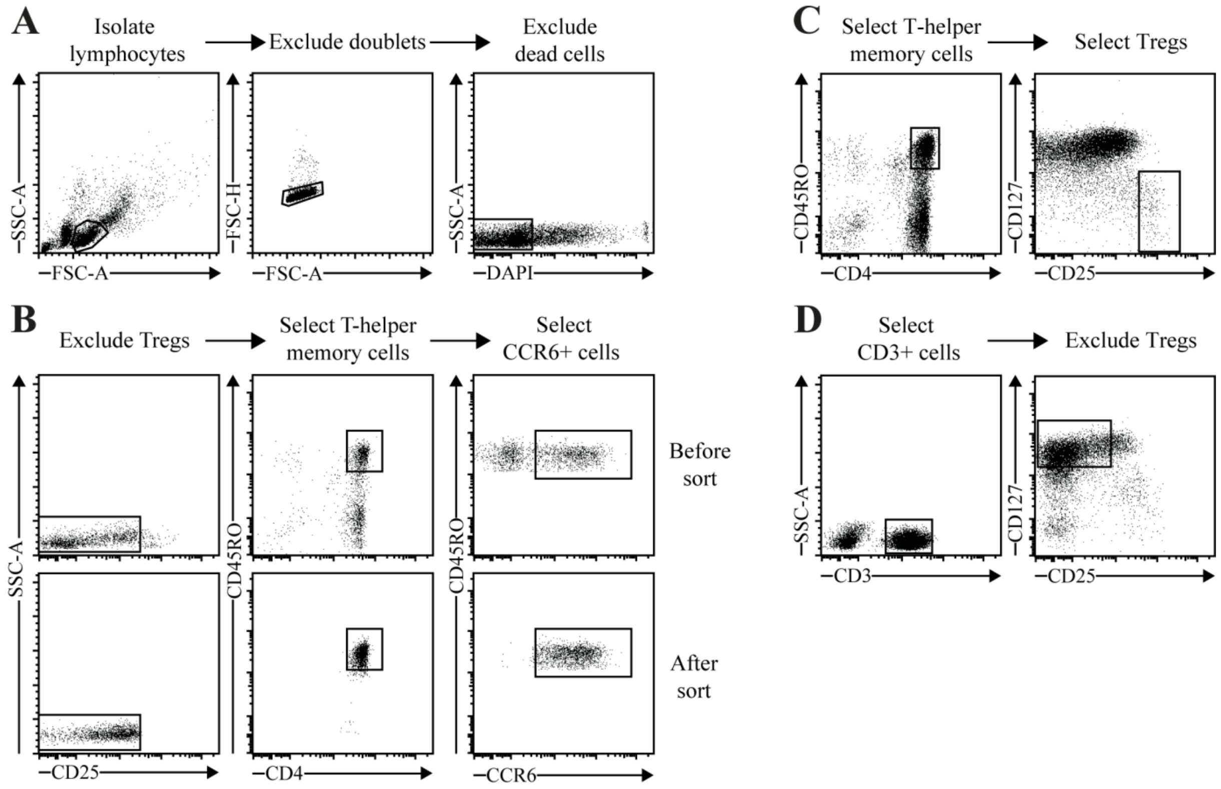

Figure S1. Typical sorting strategy from frozen PBMC. (A) Backbone gating, with lymphocyte selection, doublet exclusion and dead cell exclusion. (B) Proceeding from (A), to sort CCR6+ Th memory cells. Examples are given before sort (top panels) and after sort (bottom panels) to check for purity and exclusion of Tregs. (C) Proceeding from (A) to sort Tregs. (D) Proceeding from (A) to sort responder cells for suppression assays; CD3+ T cells excluding Tregs.
