# Supplementary material for: Human Memory Th17 Cell Populations Change Into Anti-inflammatory Cells With Regulatory Capacity Upon Exposure to Active Vitamin D
Source: Front Immunol. 2019 Jul 17;10:1504. doi: 10.3389/fimmu.2019.01504 (PMC6651215; doi:10.3389/fimmu.2019.01504)
Supplement: Supplementary file 5 [file Image_2.pdf]

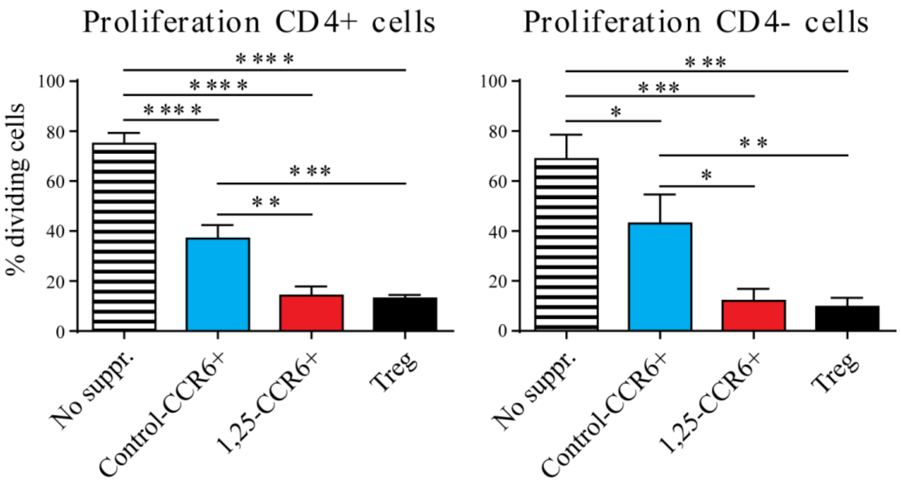

Figure S2. 1,25$(OH)_2D_3$-treated CCR6+ Th memory cells equally suppress proliferation in CD4+ and CD4- cells. Suppression assay is set-up as in figure 2, but during analysis of proliferation, responder cells are distinguished based on CD4 expression. Mean and SEM represent 3 healthy donors. Data are representative of 4 independent experiments. *p<0.05, **p<0.01, ***p<0.001, ****p<0.0001.
