# Supplementary material for: Human Memory Th17 Cell Populations Change Into Anti-inflammatory Cells With Regulatory Capacity Upon Exposure to Active Vitamin D
Source: Front Immunol. 2019 Jul 17;10:1504. doi: 10.3389/fimmu.2019.01504 (PMC6651215; doi:10.3389/fimmu.2019.01504)
Supplement: Supplementary file 6 [file Image_3.pdf]

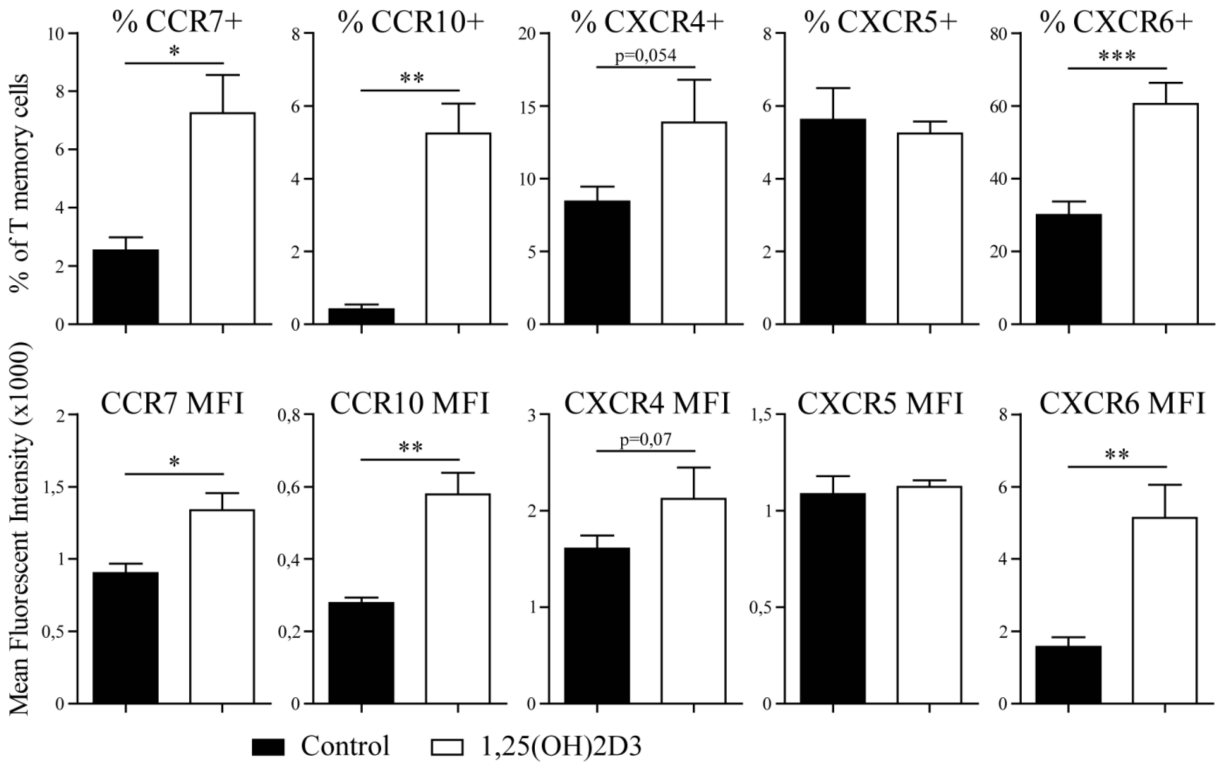


**Figure S3.** 1,25$(OH)_2D_3$-treated CCR6+ Th memory cell express a different chemokine receptor profile compared to untreated cells. Cells were sorted and cultured as in figure 3. Chemokine receptor expression was quantified using flow cytometry. Mean and SEM are given for 6 healthy donors. *p<0.05, **p<0.01, ***p<0.001.
